# Supplementary material for: NOJAH: NOt Just Another Heatmap for genome-wide cluster analysis
Source: PLoS One. 2019 Mar 28;14(3):e0204542. doi: 10.1371/journal.pone.0204542 (PMC6438523; doi:10.1371/journal.pone.0204542)
Supplement: S1 File — (DOCX) [file pone.0204542.s001.docx]

## Supplementary Material

## Data Availability

### **Example CoMMpass data**

The Multiple Myeloma Research Foundation (MMRF) CoMMpass (Relating Clinical Outcomes in Multiple Myeloma to Personal Assessment of Genomic Profile) trial (NCT 01454297), interim analysis (IA9) release was downloaded from the MMRF researcher gateway portal (<https://resaerch.themmrf.org>). A total of 560 patients with RNA-Seq gene level expression, Copy Number Variation (CNV), Variant allelic frequency, translocations and clinical data were extracted. The raw HTSeq counts from gene level RNA-Seq were DESeq normalized [1] and log2 (count +1) transformed. A variant proportion for each gene variant for each sample were downloaded from the from the exome sequencing assay and transformed using log2 ((1+p)/(1-p)). Copy segment mean values were downloaded from the long-insert whole genome sequencing assay and transformed by adding the lowest segment mean value among all the samples to each copy segment. Additionally, patient cytogenetics was obtained from the MMRF data portal’s ‘Analysis Tools’ section and then divided into two subgroups: patients reported with translocation t(11;14) or t(14;16) or t(14:20) or del17 or p53 abnormalities were classified as High Risk (HR) (n=135) whereas the remaining that didn’t qualify the HR condition were classified as Not High Risk (notHR) (n= 425). Heatmap analysis workflows based on the coMMpass data have been illustrated in this supplementary section.

## Methods

### **Genome-Wide Heatmap (GWH) Analysis**

### **Define the Core Gene-set using the Most Variable Features Approach**

NOJAH allows the user to choose the ‘Core genes’ set based on three different criteria: (a) distribution of the data itself, (b) filtering methods that are most suited to their data: VAR, MAD, and IQR or their combinations and/or (c) inclusion or exclusion of a gene within the gene-set.

The row-wise measures of spread i.e. the filters for defining most variable genes are calculated using four methods:

**Variance:** ${VAR}_{i}= \frac{\sum{(X_{i, j}-\bar{X_{i}})}^{2}}{N_{i}-1}$

**Median Absolute Deviation:** ${MAD}_{i}=median (\left| X_{i,j}-median\left( X_{i} \right) \right|)$

**Inter-Quantile Range:** ${IQR}_{i}= Q_{3 (i)}- Q_{1 (i)}$

where i = 1, 2, …., G rows and j = 1, 2, …., S samples. Q_1_ and Q_3_ represent

the first and third quantile respectively.

**Integrated Most Variable Analysis (IMVA)**

${IMVA}_{i}={\mathrm{rank} \left( {VAR}_{1,\ldots G} \right)}_{i}+{rank\left( {MAD}_{1, \ldots G} \right)}_{i}+{rank\left( {IQR}_{1, \ldots.G} \right)}_{i}$or a combination of any two ranks.

Ranks indicate the position of the specific measure among all values within the measure. For example, the position for the variance of a specific gene at position i is calculated in comparison to all row-wise variances i.e. VAR_(1,...., G)_. Genes are ranked using the rank function in R and values with ties are sorted based on the minimum method. Similarly, the gene ranks for MAD and IQR is calculated. The sum of ranks is the IMVA statistic.

A boxplot representing the row-wise spread for the VAR, MAD and IQR are generated to help choose the appropriate filters. Filters that show a larger spread are preferred. Users can choose the ‘Core Genes’ using a single or a combination of filters. By default, a VAR is selected. Furthermore, rather a percentile cut-off can be applied to filter out the genes that are less than the desired cut-off, for example, using a 99^th^ percentile cut-off for the VAR statistic, the top 1% of the data is selected. This subset data is available for download in the left panel.

### **Heatmap with Core Genes**

An interactive heatmap is automatically generated for the user-selected most variable feature subset under the ‘Heatmap’ Tab. Heatmap is created, based on a modified *heatmap.2* function (<https://github.com/obigriffith/biostar-tutorials/blob/master/Heatmaps/heatmap.3.R>). Users can easily modify and compare the heatmap(s) using a simple point and click functionality. They can choose between three scaling methods: based on Z-score or a modified Z-score or none, among three normalization methods: row, column or both row and column; and eight different most widely used distance methods: euclidean, maximum, manhattan, canberra, binary, minkowski (available in *heatmap.2* function) or additionally the 1-Pearson correlation distance. The users also have a choice between the eight different agglomerative linkage methods: average, complete, ward.D, ward.D2, single, mcquitty, median or centroid (available as a part of the *heatmap.2* function (underlined being the defaults)). After each parameter is changed, the user should hit the ‘Run Analysis’ button to display the changes to the heatmap.

Scaling and data normalization is computed as follows:

z-score $Z_{i}= \frac{X_{i}- \bar{X}}{s}$

Modified z-score ${modZ}_{i}= \frac{(X_{i}-median \left( X_{i} \right))}{1.253314*MeanAD}$ when MAD = 0

$= \frac{(X_{i}-median \left( X_{i} \right))}{1.486*{mad}_{i}}$ when MAD ≠ 0

where i = 1, …., G rows. Column-wise scores can be calculated by transposing datasets. Both rows and column z-scores are calculated by first calculating row-wise z-scores and then column wise z-scores for the already calculated row-wise z-scores.

Data is scaled before input into the modified heatmap.2 function. The row and column dendrograms are displayed using the *hclust* function of the same package. By default, both the row and column dendrograms are reordered based on the means. An option is available to the user to instead sort the samples based on the user-defined groups (by selecting ‘column dendrogram’ = false). The sample and gene labels can be displayed at the bottom and on the right side respectively, using the appropriate options and their font sizes can be increased or decreased using the sliders. Based on the type of data, the user can choose an appropriate color scheme for the heatmap. For example, here the expression data is represented in green-black-red. When using methylation data, the colors can be changed to blue-white-red to represent low-mid-high expression respectively. Additional details about the options are available in the tutorial tab.

The column and row dendrograms are also extracted and displayed in separate tabs. The user can further cut the row and/or column dendrogram trees into the desired number of clusters using the cut tree function of the ‘stats’ package. The default has been set for two clusters and can be changed depending on the user input. The classification of samples and their clusters is available for download as a CSV file for analysis outside of NOJAH.

Within the Heatmap tab, the user has a choice to upload their own subset data. They can do so using the ’Subset Heatmap Input’ drop-down on the top left corner and select the load my own data option. When this option is selected, additional information such as the exact row and column where the numeric data starts needs to be provided. For example, in the example TCGA BRCA Expression data, numeric data starts on the row number three (first and second row contain sample names and phenotype information respectively) and column number three (first and second columns being gene_id and Groups respectively). This is especially important in cases where additional sample information is appended in a separate row(s) below the main phenotype groupings and just above the numeric data. Additional information about the genes are included to the right of the row groups (‘Groups’ column). Any missing information within these additional rows or columns should be marked as none. NA’s or blanks may cause the program to display errors (see NOJAH homepage for details). If this additional column/row is labeled, this appears beside the corresponding bar on the heatmap. All other functionalities are similar as described in the example data.

The output from the heatmap tab is a PDF file with the minimal information required to reproduce it such as the scaling, normalization, clustering and distance measures. Additionally, the column and row dendrogram are also provided in the PDF file.

### **Define Cluster Number**

Using either the ‘Core Genes’ or a user-provided gene set, the number of clusters within a dataset can be estimated with quantitative evidence based on the ConsensusClusterPlus Bioconductor package [2]. A few parameters available are a choice of 10/100/500/1000 iterations (100 being default) and the number of optimal clusters (10 being default). Furthermore, a choice between the seven distance measures and eight different clustering methods (as available in the Heatmap Tab) are available. During each run 80% sample resampling with the proportion of gene resampling equal to 1 is performed. The output from the ConsensusClusterPlus Bioconductor package includes a consensus cluster heatmaps for each cluster ‘k’, along with a CDF plot which helps determine the optimal k at which the distribution reaches maximum stability. Also, a delta area plot is provided which represents the change in the area under the CDF curves. The largest k at which there is an appreciable increase in consensus is selected as the optimal k. Please see [2] for additional details .

The user can use this tab independently to identify the optimal number of clusters within a gene-subset of their choice (done outside of NOJAH or using other settings within NOJAH). This feature is made available using “Load your own dataset” within the Consensus Clustering Input section in the top left corner. The subset input data file should be in the same format as that of the example data (available for download on the left panel).

### **Define Core Samples**

Once the optimal number of clusters are identified, the validity of the consistency within the clusters can be assessed visually using a silhouette plot [3]. This method compares how close a sample is to other samples in its own cluster with how close it is to samples in other clusters using a metric called the silhouette width.

Silhouette width is computed for each observation i.e. sample, j as

$$S\left( i \right)= \frac{b_{(j)}-a_{(j)}}{max(a_{(j)}, b_{(j)})}$$

where $a_{(j)}$= average dissimilarity with members of the cluster to which j belongs and $b_{(j)}$= minimum average dissimilarity to members of another cluster.

In the ‘Core Samples’ tab, silhouette width is estimated using the silhouette function from the ‘cluster’ R package. Larger silhouette width values (near one) means that the samples are well placed vs. those near zero, meaning that fit was not good. Thus, samples with negative silhouette widths indicate an extremely poor fit and these samples could be dropped from that cluster. The remaining samples compose the ‘core’ sample set.

Within NOJAH, the core samples can be selected based on three different criteria: separate (a) silhouette width cut-off (b) percentile cut-off and (c) change point, for each cluster. In case of silhouette width cut-off (a), only those samples which exceed a user-specified silhouette width (based on each separate cluster) are extracted as the ‘core sample’ set. Similarly, when percentile cut-off (b) is chosen, the user can choose whether to remove the bottom p^th^ percentile of the samples within each cluster. A more data-driven approach is to use a change point model (c) to filter out that sample that does not meet the desired change point cut-off (for example, a stringent cut-off would be including only those samples which are included in change point one) [4]. To estimate a stable cut-point within each cluster, each cluster should include at least 5 samples. Alternately, the user can also upload a dataset with their own clusters (in the same format as the example data) to identify the core samples based on either criterion.

Once the core sample set is selected, NOJAH automatically creates another interactive Heatmap in the ‘Updated Heatmap’ tab, based on these ‘Core Genes with Core Samples’ and all the interactive features like the previously generated heatmap (see GWH part 2).

NOJAH additionally displays the exact settings used in the GWH workflow in the ‘Workflow’ tab. Using these settings, the user can easily replicate the analysis. This workflow can be saved on the user’s local computer using a ‘snipping tool’ for windows users or the grab tool for Mac OS users.

### **Combined results Clustering (CrC) Analysis**

Using NOJAH’s CrC analysis tab, a user can combine clustering results from up to three different data types, for example, expression, methylation, copy number and/or variant allelic frequency. Alternately, users can choose to input their own clusters (done outside of NOJAH using, for example, NMF clustering, k-means, or using other heatmap analysis tools). If the user chooses to cluster their data within NOJAH, data from each data type can be clustered separately using a first level consensus clustering and then these clustering results are automatically carried forward and combined to the 1-0 matrix for a second-level consensus clustering performed in the ‘Cluster of Cluster (CoC)’ analysis tab. Alternately, if the user chooses to input their own clusters, a single data file with the samples as columns and the rows with each data type include the cluster numbers should be input as a CSV file in the Cluster of Cluster analysis tab. All interactive features from the heatmap tab of the GWH workflow are also available. The consensus clustering results as PDF files are available for download in each case. When in the CoC tab, additionally, a PDF with the heatmap and minimal information to reproduce it is also provided.

When the actual data on each data type is supplied into NOJAH for clustering, in addition to the heatmap, boxplots are made available which suggest how individual cluster fair among each data type. For example, Expression E1 has higher median expression than E2. Boxplots are displayed only when the entire CrC pipeline from NOJAH is used. If using your own clusters in the cluster of cluster analysis tab, interpretation plots are not available to the user due to the lack of the actual data points on which these plots are based. In addition to the boxplots which aid interpretation of the clusters, the distribution of samples within each cluster are also available as contingency tables. When all three data types are used, the contingency tables are stratified by the user provided data type i.e. Expression, Methylation/Variant or CNV.

### **The Significance of Cluster (SoC) Analysis**

SoC analysis is based on a monte-carlo bootstrap approach. At least two sample groups are required to perform SoC analysis. Based on the user provided data i.e. observed data, a fisher’s exact test is performed to test if the clustering can differentiate the sample groups. A significant association between the clusters and sample groups is desirable. Only if this association is significant, SoC analysis is performed.

SoC analysis is iterative process. Genome-wide data with the same exact samples and groupings as the observed data needed to be provided as input. First the user chooses to randomly sample a gene-set of the same size to their observed data from the genome-wide data. Second, a heatmap clustering analysis using the same settings as the observed data is performed on this new dataset. Third, a Fisher’s exact test is performed between the new clusters and the phenotypes and a p-value is computed. Steps 1-3 are repeated several times based on the user input. Many iterations up to 1000 times is preferred. A ‘Go’ button is available to initiate the process.

The output from the analysis is a p-value which denotes the number of times the permuted p-value was greater than the observed p-value. If significant, this denotes that the gene set of interest can separate sample groups, outperforming n random samples of genes of the same number in this regard (n being the number of iterations chosen by user). A tabular output is appended to the heatmap PDF output. Similar analysis can be conducted for the row/gene groups.

## Data Analysis using the CoMMpass dataset

GWH Analysis

Using the genome-wide coMMpass IA9 RNASeq gene expression data from the 560 multiple myeloma patients, the GWH workflow is implemented. The column dendogram (Figure A) based on 54,592 genes expression using z-score, row normalization with 1-pearson correlation and average agglomerative linkage suggests the presence of at least five potential clusters. Based on the boxplots for the three measures of spread (Figure A), the VAR (variance) shows a larger spread and is used to define a topmost variable i.e. core gene set by extracting genes with a VAR above 97.5^th^, resulting in 1450 core genes. A heatmap of this core gene set shows a visual separation of samples into at least five clusters as compared to the genome-wide dendogram (Figure A). Consensus clustering based on 1-pearson correlation, average clustering, 100 iterations, 80% item resampling, 80% gene sampling, and agglomerative hierarchical clustering algorithm confirms the presence of at least five clusters (Figure A). Silhouette plot shows a few samples within each cluster with low widths and after their removal (silhouette width with less than 0.1 in cluster 1 and 0 in cluster 2-5), results in tighter clusters with 111, 100, 99, 90, and 94 respectively (Figure A) . Using these 494 core samples with the consensus cluster classification, the heatmap is updated for the core samples (Figure A). The original sample classification is available as a color bar over the consensus clusters. The updated heatmap is created using z-score normalization, both row and column scaling, euclidean distance and average clustering. Overall, the total time elapsed to perform this type of GWH analysis of RNA-Seq derived gene expression data on the 560 multiple myeloma patients was less than 2.5 minutes using the computational configuration outlined in Figure B.

**
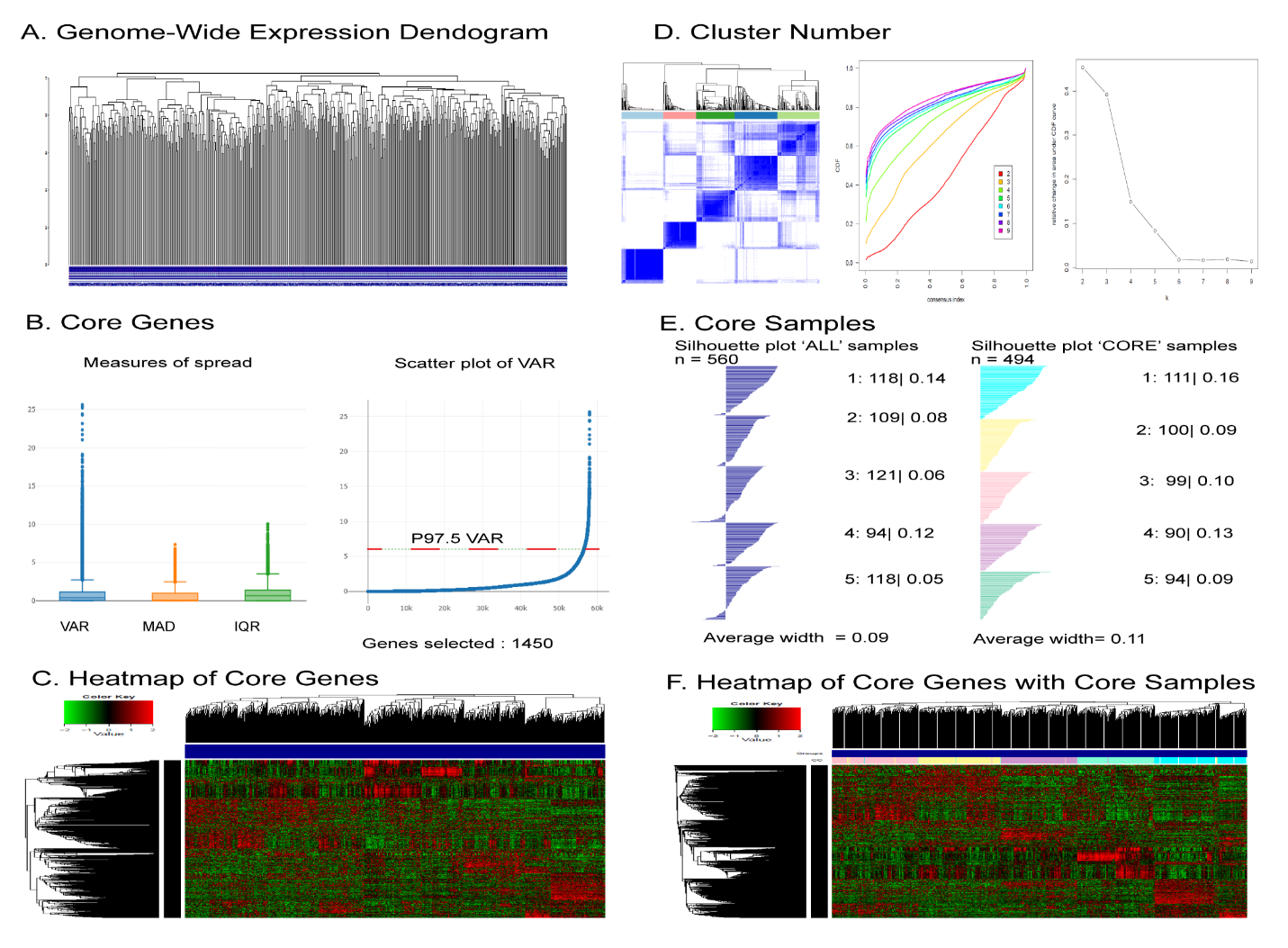
 Figure A. NOJAH genome-wide heatmap (GWH) of RNA-Seq derived gene expression data using CoMMpass expression dataset.** A. Genome-Wide Expression Dendrogram based on 54592 genes showing at least three clusters using row normalization, 1-person correlation distance and average clustering B. *Defining Core Genes.* Distributions of measures of spread, VAR (variance), MAD (median absolute deviation), and IQR (inter-quartile range) showing IQR as most variable showing VAR is as most variable. An ordered VAR values for each gene shows 97.5^th^ percentile as a cut point to define 1450 topmost variable genes (i.e. ‘core gene set’). C. *Heatmap of Core Genes.* Heatmap using core gene set with options: z-score based row normalization, 1-pearson correlation distance, and agglomerative average linkage shows five gene and sample clusters. D. *Defining Number of Clusters*. Results from consensus clustering using 1-pearson correlation distance, average clustering, 80% item resampling, 100% gene sampling, and agglomerative hierarchical clustering shows five clusters. E. *Defining Core Samples*. Silhouette plots of samples within each of five clusters. Samples with a silhouette-width less than 0.1 in the first and 0 in the remaining clusters were removed to define the ‘Core subset’ of 111, 100, 99, 90, and 94 samples respectively. *F. Heatmap of Core Genes with Core Samples.* Updated heatmap with options: row and column z-score normalization, euclidean distance, and agglomerative average linkage clustering based on core genes with core samples displaying distinct gene as well as sample clusters.

**
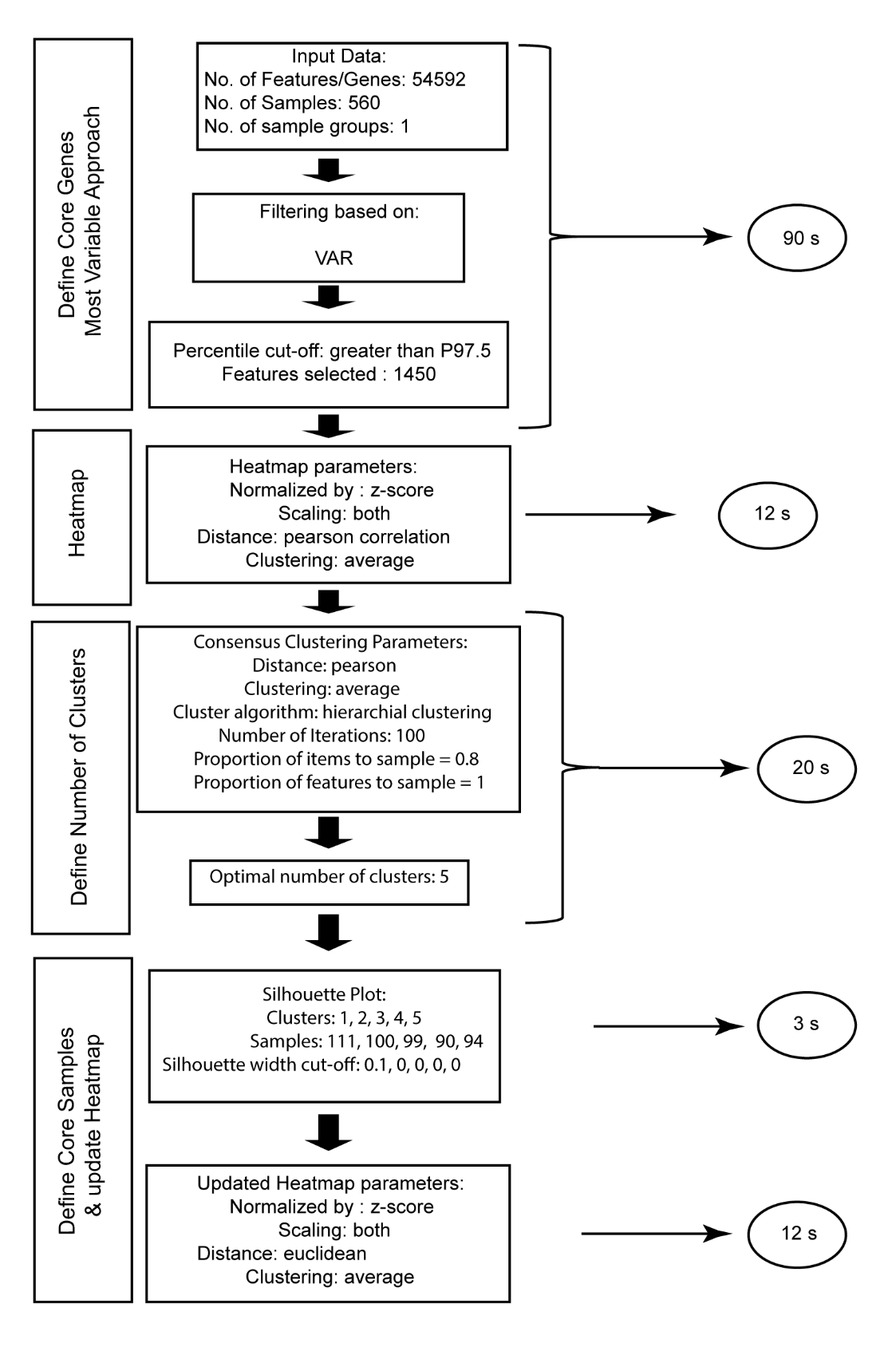
**

**Figure B. NOJAH genome-wide Heatmap (GWH) analysis output workflow using CoMMpass expression dataset.** The optional parameters used to generate each analysis case in the GWH analysis workflow are defined as part of the output. The time in seconds (s) to run each analysis case is shown in a circle. The total time elapsed to perform a GWH analysis of RNA-Seq derived gene expression data using our example of 560 multiple myeloma cancer patients was less than 2.5 minutes when run on a local Windows 10 Enterprise machine with Intel(R) Core ™ i-7-7820HQ CPU 2.90GHz and 32GB RAM.

**CrC Analysis**

A GWH analysis workflow was applied separately on each of the RNA-Seq derived gene expression, variant proportion and copy number data on the 560 multiple myeloma patients. Within each data type, a core gene set was defined (Figure C). For gene expression and variant proportion data, VAR was used to define topmost variable 1450 core genes and 1512 core variants, based on the 97.5^th^ and 99.1^st^ percentile respectively. In case of copy number data, MAD was used based on the 97.5^th^ percentile and 1447 topmost variable copy segments were extracted.

For each data type, consensus clustering was performed to define number of clusters, resulting in 5, 2, and 2 optimal clusters for expression, variant and copy number respectively (Figure C). Expression clusters were predicted using 1-pearson correlation distance, variant clusters using maximum distance and copy number clusters using manhattan distance. Average agglomerative clustering was used in each data type with 50 iterations, 80% item resampling, and 100 % gene sampling. Silhouette plots show good the homogeneity of the clusters within each tab (data not shown). Cluster classification from each data type is combined into a 1-0 matrix and consensus clustering analysis is performed on it. Two optimal number of clusters are predicted using the ConsensusClusterPlus package using similar parameters like before except euclidean distance, average clustering and 100 iterations. Based on the CrC heatmap (Figure C), cluster 2 (CrC 2 in red), includes higher proportion of E2, E4, E5, V1, and CNV2 whereas cluster 1 (CrC 1 in blue) includes a mixture of samples with higher proportion of samples from E1, E3, V2 and CNV1. Boxplots of sample groups by data type (Figure C) suggests that CrC 1 includes samples with increased expression, increased variants and decreased copy number segments whereas CrC 2 with decreased expression, decreased variants and increased copy number. A contingency table when stratified by variant clusters shows that there is a significant association between expression clusters and copy number clusters within each variant cluster.

**
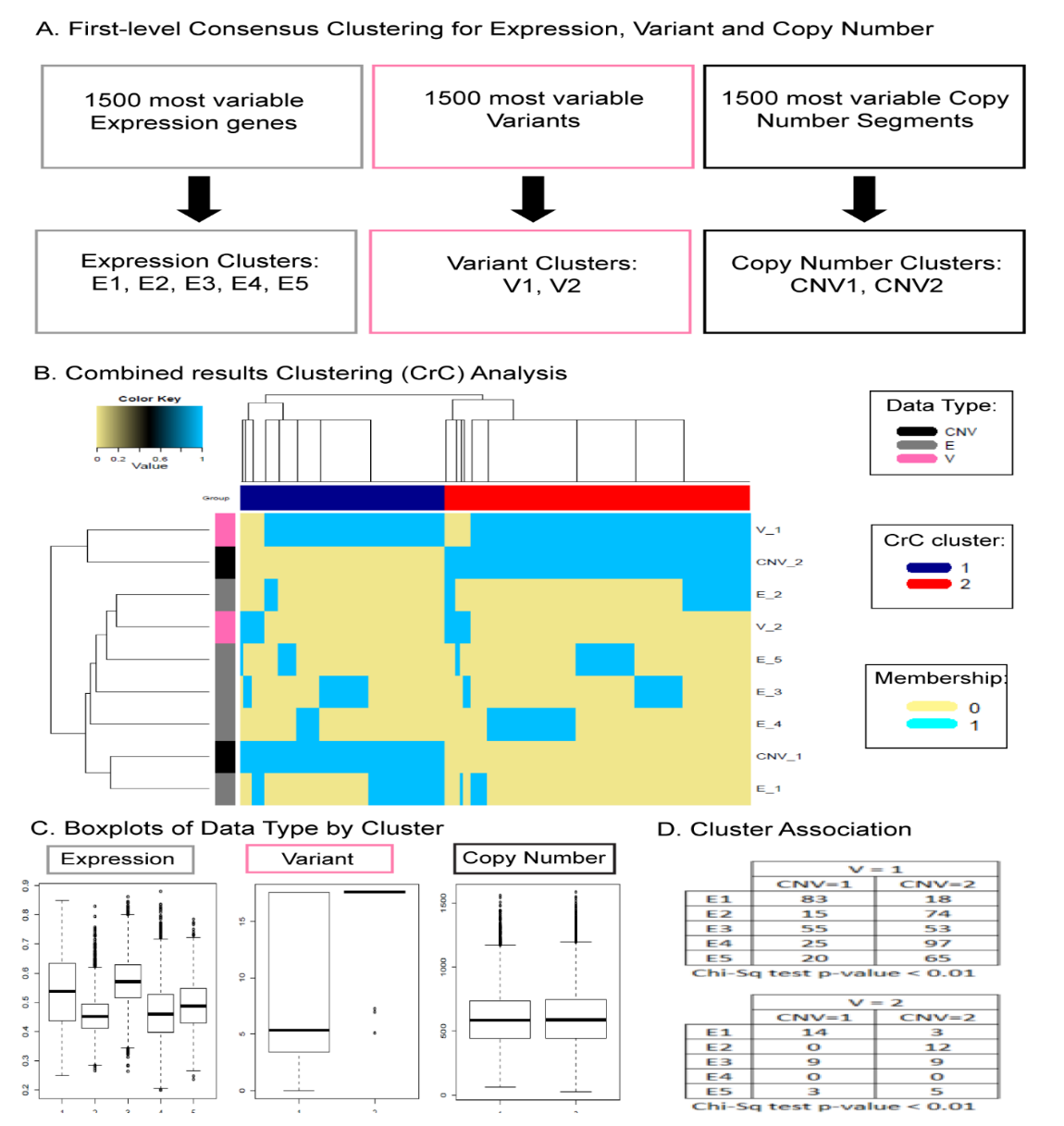
**

**Figure C.** **NOJAH combined results clustering (CrC) analysis of gene expression, variant proportion and copy number data using CoMMpass dataset.** A) *Genome-wide Heatmap (GWH) Analysis.* A GWH analysis workflow was applied to each data type, resulting in 5, 2, and 2 sample clusters based on defined most variable genes, variants and copy number segments. B) *Heatmap of cluster results*. Using a binary (0-1) matrix to indicate sample cluster membership based on individual data types, a heatmap shows two sample clusters that was also indicated by consensus clustering. One cluster (CrC 2 in red) includes samples from predominantly CNV2, V1 but also E2, E4, E5, clusters. The other cluster (CrC 1 in blue) includes mainly CNV1 but also a mixture of samples from the various clusters. C) *Cluster Interpretation*. Boxplots of data type clusters indicated that CrC 2 includes samples with decreased variants (V1), increased copy number (CNV2) as well as those with relatively low expression (E2, E4 and E5). A mixture of samples defines the CrC 1 cluster, including those with increased variants (V2), decreased copy number (CNV1) and increased expression (E1 and E3). Contingency table by variants clusters show a significant association between expression and copy number clusters.

**SoC Analysis**

Using the 1450 core genes and 494 core samples defined from the GWH analysis of the 560 CoMMpass expression samples data set in S1F Figure, five sample groups are defined. The default parameters of euclidean distance and average clustering are used. Additional color bars are added above the clustering to classify patients into High Risk (HR) vs the remaining samples. Cluster 1 (in maroon) includes most HR patients as compared to the other clusters. Splitting the column dendrogram into 5 clusters, the significance of separation of clusters into the five groups was estimated using a bootstrap approach with the genome-wide coMMpass Expression data with the same 494 core samples. This core gene-set was found to be statistically significant (Figure D) as compared to a random set with the same size (i.e. approximately 1500 genes) from the genome-wide gene expression data set, using the Monte Carlo bootstrap approach and 1000 iterations, in separation of the five sample groups (p-value < 0.01).

**
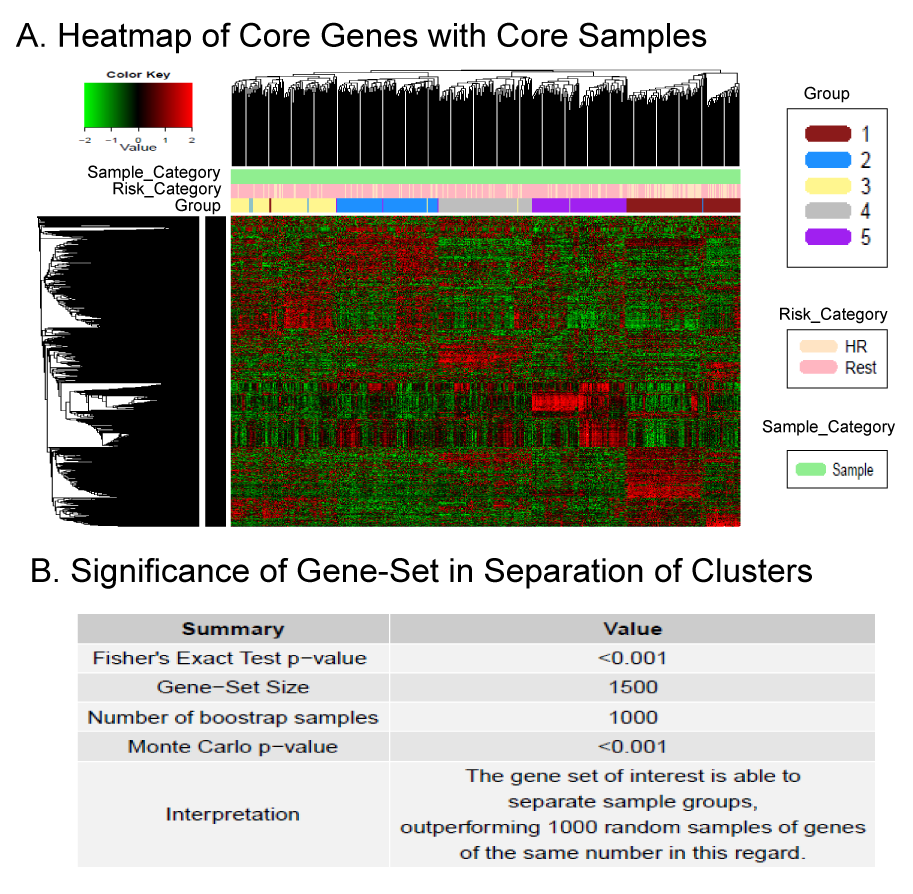
**

**Figure D. NOJAH significance of cluster (SoC) analysis on core gene set with core sample CoMMpass dataset.** A. NOJAH generated heatmap using 1450 Core Genes and 494 Core samples from the GWH analysis. Z-score scaling for both row and column, euclidean distance with agglomerative average linkage shows clear separation of cluster groups; Group1(n= 111, in maroon), Group2 (n = 100, in blue), Group3 (n =99, in yellow), Group4 (n= 90, in grey) and Group5 (n= 94, in purple). Most HR patients are clustered in cluster 1 (in light orange color above maroon cluster). B. Estimation of p-values to assess the significance of a random gene set in the separation of clusters into the five groups by Fisher's exact test is statistically significant (p <0.01).

## References:

1. Love MI, Huber W, Anders S. Moderated estimation of fold change and dispersion for RNA-seq data with DESeq2. Genome Biol. 2014;15(12):550. Epub 2014/12/18. doi: 10.1186/s13059-014-0550-8. PubMed PMID: 25516281; PubMed Central PMCID: PMCPMC4302049.

2. Wilkerson MD, Hayes DN. ConsensusClusterPlus: a class discovery tool with confidence assessments and item tracking. Bioinformatics. 2010;26(12):1572-3. Epub 2010/04/30. doi: 10.1093/bioinformatics/btq170. PubMed PMID: 20427518; PubMed Central PMCID: PMCPMC2881355.

3. Rousseeuw PJ. Silhouettes - a Graphical Aid to the Interpretation and Validation of Cluster-Analysis. J Comput Appl Math. 1987;20:53-65. doi: Doi 10.1016/0377-0427(87)90125-7. PubMed PMID: WOS:A1987L111800005.

4. Dwivedi B, Kowalski J. shinyGISPA: A web application for characterizing phenotype by gene sets using multiple omics data combinations. PLoS One. 2018;13(2):e0192563. Epub 2018/02/08. doi: 10.1371/journal.pone.0192563. PubMed PMID: 29415010; PubMed Central PMCID: PMCPMC5802933.
